# Individualized 3D-Printed Tissue Retraction Devices for Head and Neck Radiotherapy

**DOI:** 10.3389/fonc.2021.628743

**Published:** 2021-03-23

**Authors:** Christopher Herpel, Franz Sebastian Schwindling, Thomas Held, Leo Christ, Kristin Lang, Martha Schwindling, Julius Moratin, Karim Zaoui, Tracy Moutsis, Peter Plinkert, Klaus Herfarth, Christian Freudlsperger, Peter Rammelsberg, Jürgen Debus, Sebastian Adeberg

**Affiliations:** ^1^ Department of Prosthetic Dentistry, Heidelberg University Hospital, Heidelberg, Germany; ^2^ Department of Radiation Oncology, Heidelberg University Hospital, Heidelberg, Germany; ^3^ Heidelberg Institute of Radiation Oncology (HIRO), University Hospital Heidelberg, Heidelberg, Germany; ^4^ National Center for Tumor Diseases (NCT), University Hospital Heidelberg, Heidelberg, Germany; ^5^ Design Studio M. Schwindling, Berlin, Germany; ^6^ Department of Oral and Maxillofacial Surgery, Heidelberg University Hospital, Heidelberg, Germany; ^7^ Department of Otorhinolaryngology, University of Heidelberg, Heidelberg, Germany; ^8^ Heidelberg Ion-Beam Therapy Center (HIT), University Hospital Heidelberg, Heidelberg, Germany; ^9^ Clinical Cooperation Unit Radiation Oncology, German Cancer Research Center (DKFZ), Heidelberg, Germany; ^10^ German Cancer Consortium (DKTK), partner site Heidelberg, German Cancer Research Center (DKFZ), Heidelberg, Germany

**Keywords:** HNSCC, advances in management, 3D printing, tissue retraction, radiation therapy, oral stents, tongue displacement, intraoral splints

## Abstract

**Background:**

Radiotherapy for head and neck cancer may cause various oral sequelae, such as radiation-induced mucositis. To protect healthy tissue from irradiation, intraoral devices can be used. Current tissue retraction devices (TRDs) have to be either individually manufactured at considerable cost and time expenditure or they are limited in their variability. In this context, a 3D-printed, tooth-borne TRD might further facilitate clinical use.

**Methods:**

A novel approach for the manufacturing of TRDs is described and its clinical application is analysed retrospectively. The devices were virtually designed for fabrication by 3D-printing technology, enabling—in only a single printing design—caudal or bi-lateral tongue displacement, as well as stabilization of a tongue-out position. For a total of 10 patients undergoing radiotherapy of head and neck tumors, the devices were individually adapted after pre-fabrication. Technical and clinical feasibility was assessed along with patient adherence. Tissue spacing was calculated by volumetric analysis of tongue retraction. In one exemplary case, radiotherapy treatment plans before and after tissue displacement were generated and compared. The reproducibility of maxillomandibular relation at device re-positioning was quantified by repeated intraoral optical scanning in a voluntary participant.

**Results:**

3D-printing was useful for the simplification of TRD manufacture, resulting in a total patient treatment time of less than 30 min. The devices were tolerated well by all tested patients over the entire radiation treatment period. No technical complications occurred with the devices. The TRDs caused an effective spacing of the healthy adjacent tissue, e.g., the tongue. Position changes of maxillomandibular relation were limited to a mean value of 98.1 µm ± 29.4 µm root mean square deviation between initial reference and follow-up positions.

**Conclusions:**

The presented method allows a resource-efficient fabrication of individualized, tooth-bourne TRDs. A high reproducibility of maxillomandibular relation was found and the first clinical experiences underline the high potential of such devices for radiotherapy in the head and neck area.

## Introduction

Radiotherapy plays a key role in the treatment of head and neck tumors (1). During and after radiation treatment, intraoral sequelae, e.g. radiation-induced oral mucositis (RIOM), can occur. Higher-grade RIOM occurs in up to 60% of the patients receiving head/neck radiotherapy (2). It can lead to pain, ageusia, superinfection, dysphagia, and weight loss (3).

Tissue retraction devices (TRD) increase the distance between tumor and healthy tissue, with potential consequences on the prevalence and severity of RIOM (4, 5). Even small geometric changes can lead to significantly less irradiation of healthy tissue and can, thereby, significantly reduce side effects (6–8). The manufacture of TRDs is, however, complex. Traditionally, dental impressions are taken, and stone models are poured. Then TRDs are sculpted from wax and transferred into acrylic resin (9).

Novel computer-assisted design and manufacturing (CAD/CAM) techniques might help to increase TRD quality, simplify the workflow, and reduce manufacturing costs. Even though the evidence on CAD/CAM-based TRDs is still limited, first results are promising. A significant decrease in radiation dose to the tongue was demonstrated using 3D-printed devices (10). Kitamori et al. suggested advantages of 3D-printed TRDs in terms of dose distribution with reduction of the integral dose to the surrounding normal tissue (11). Additionally, scattered radiation by dental restorative metals might be effectively absorbed by 3D-printing resin (11).

Apart from increasing the distance between tumor and healthy tissue in order to reduce RIOM, TRDs might also be advantageous for accurate re-positioning of the patient. Ensuring positional consistency between treatment days is an important goal in head and neck radiotherapy (12). Given an adequate design, 3D-printed TRDs might support the accurate interfractional patient setup by using the remaining dentition for a rigid inter-jaw fixation, thus providing a defined position of the lower jaw in relation to the upper jaw. This might reduce longitudinal deviations in maxillomandibular relation (11).

The clinical application of novel CAD/CAM-based TRDs was assessed in an individual approach in 10 patients undergoing radiotherapy of head and neck tumors. These devices are not limited to either tongue depression (11, 13) or tongue lateralization (10), but they allow—in only a single printing design—caudal or bi-lateral tongue displacement, and stabilization of a tongue-out position. In this study, tongue retraction was quantified using volumetric analysis of the irradiation plans. The reproducibility of maxillomandibular relation was quantified by repeated intraoral optical scanning.

## Materials and Equipment

**Figure 1:**
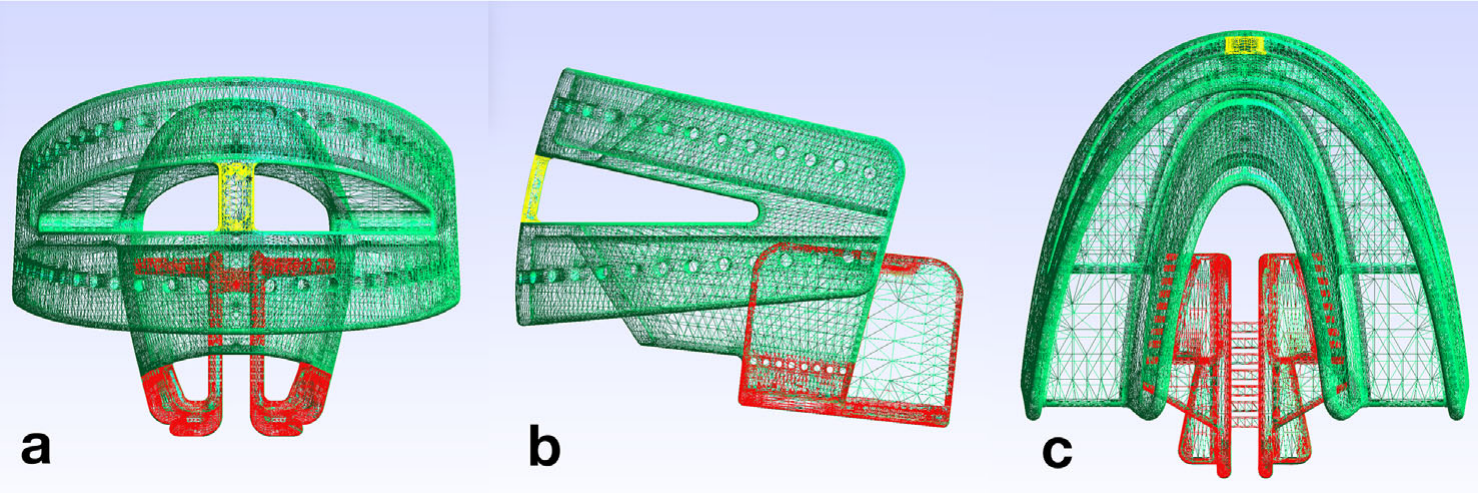
Design file of the tissue retraction device; the fixation part (FP) is shown in green and the tongue retraction part (TP) in red. The connection bar (yellow) bridges upper and lower parts of the TRD. **(A)** front view, **(B)** lateral view, **(C)** top view.

A new design of CAD/CAM-based TRDs for head and neck radiotherapy was virtually designed (Rhinoceros 3D) for fabrication with 3D-printing technology (**Figure 1**).

Key design characteristics are described in **Table 1**. Using 3D-printing, the TRDs are pre-fabricated in three sizes (small, medium, large in accordance with the common dental impression tray sizes), and can be stocked in advance. The 3D-printed TRDs are adapted to the individual patient as soon as the type of tissue displacement has been specified. The TRDs consist of a fixation part (FP, marked green in **Figure 1**) and a tongue retraction part (TP, marked red in **Figure 1**). The fixation part encloses the remaining teeth similar to a dental impression tray. The tongue retraction part controls tissue displacement and can be removed in part or completely, depending on the irradiation plan. For a caudal displacement, the framework is kept in its complete integrity. For tongue lateralization to the right side, the right part of the tongue retraction part is removed and vice versa for left side. To achieve a tongue-out position (6), the entire tongue retraction part is removed. At the most anterior point of the TRD, a connection bar bridges upper and lower fixation parts (marked yellow in **Figure 1**) To stabilize the tongue-out position, the patient is instructed to keep the tip of the tongue in direct contact with this bar during the entire radiation session. As this position is not over-extended, it should be viable for the patient to maintain without considerable discomfort. To produce a stock of TRDs, a 3D-printer (Pro2, Asiga) was used in combination with dental splint resin (Freeprint splint 2.0, Detax). 3D-printing was performed after nesting the CAD files in a 45° building angle, in layers of 100 µm. Then, printing supports were removed and the devices were cleaned in an ultrasonic bath with 70% alcohol. Subsequently, the devices were light-cured in a xenon-flashlight curing machine.

**Table 1:** Key design characteristics of the TRD.

TRD characteristics	Aims
CAD/CAM-based production by 3D-printing	Cost-efficient manufacture, favorable dose distribution and dose-volume histogram
Fixation at the remaining teeth	Accurate patient re-positioning
Complete covering of teeth	Prevention of scattered radiation
Tongue displacement in various directions: caudal, ventral, left lateral, right lateral	Variable tissue retraction to reduce radiation dose to healthy structures
Mouth opening and mandibular protrusion	
Lip- and cheek-spacing	
Customization of pre-fabricated TRDs with silicone material, retained by perforations	Time-efficient adaptation (< 30 min)

## Methods

### Customization Procedure

**Figure 2:**
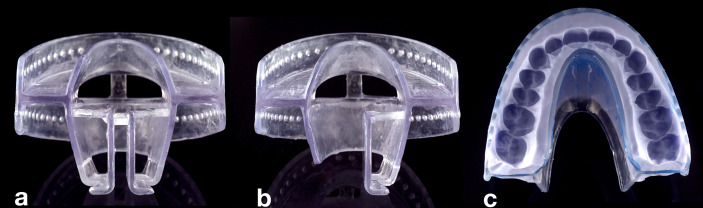
View of a pre-fabricated TRD. After size selection, different tongue displacements can be realized by removal of tongue retraction parts (TPs). **(A)** If no parts are removed, caudal tongue displacement can be achieved. **(B)** The left part of the TP was removed along defined breaking points enabling tongue displacement to the left side. **(C)** TRD (top view) after removal of TP for tongue-out position, after customization with silicone material.

For adaption to the patient’s dentition, the 3D-printed TRDs are customized. The customization procedure is a three-step process, which requires a total time of less than 30 min. First, the appropriate TRD size is selected. Selection is based on the patient’s dental arch width, similarly as for the choice of dental impression trays. Second, certain TRD resin parts are removed along defined breaking points within the design (**Figure 2**). Thereby, one of the following four different tongue displacements can be realized: caudal, bi-lateral (left or right), or ventral. Third, the fixation part is filled with a dental silicone impression material (Flexitime Putty, Kulzer), to provide a fit to the individual’s dentition. Before the silicone is set, the device is adapted to the patient’s maxilla. Then, the patient is instructed to close the mouth in a slightly protruded position, thus biting with the mandibular teeth into the silicone. After the silicone is set, the TRD, to which the silicone has adhered, is removed from the mouth and any excess silicone is cut off with a scalpel, making sure that all teeth up to cervical level are embedded in it. A layer of sealing silicone is applied to refine the silicone surfaces and ensure durability (Mucopren Silicone sealant, Kettenbach) (**Figure 2C**). The patients are instructed in how to insert and remove the device.

### Clinical Application

Ten patients were retrospectively reviewed. Selection criteria were radiation treatment for head and neck tumors and utilization of the novel CAD/CAM-based TRDs based on an individual curative decision by the treating radiation oncologist. In these 10 patients, tumors of the nasal or paranasal sinuses, oropharynx, lip and oral cavity were to be irradiated. Therefore, the main goal of the TRDs was to displace the tongue out of the high-dose radiation field. The usual thermoplastic immobilization mask for head neck radiation was adjusted with TRDs placed intraorally. Contrast-enhanced computed tomography (CT) imaging (3-mm slice thickness) was performed for irradiation planning, with incorporated TRD and immobilization mask. Contrast-enhanced T1-weighted magnetic resonance imaging (MRI) was used for image registration. Treatment planning was conducted using TomoTherapy^®^ (Accuray, Sunnyvale, U.S.A.), Syngo PT Planning version 13 (Siemens, Erlangen, Germany) or RayStation^®^ (Raysearch Laboratories, Stockholm, Sweden). Treatment was performed with intensity-modulated radiation therapy (IMRT) or particle therapy, according to the standards at our clinic. The integrity of the TRD and its correct positioning was checked before each radiation treatment. Prevalence and severity of oral mucositis was assessed at the last day of the radiotherapy cycle according to the Common Terminology Criteria for Adverse Events 4.03.

### Volumetric Analysis of Tongue Retraction

To analyze the effect of TRDs for the caudal displacement of the tongue, CT imaging data of the 10 patients were exported as DICOM files. The CTs were routinely performed for irradiation planning. The DICOM files were imported into segmentation software (DICOM to PRINT, 3D Systems, Rock Hill, U.S.A.). The air volume filling the oral cavity with the TRD placed intraorally was segmented and exported as STL file. The air volume was calculated using reverse-engineering software (Geomagic Design X, 3D Systems, Rock Hill, United States).

### Reproducibility of Maxillo-Mandibular Relation

The reproducibility of maxillomandibular relation was quantified for a fully dentate voluntary participant. TRD customization was performed as described before. Immediately after silicone curing, i.e. without having removed the device, an optical, three-dimensional, intraoral scan (reference scan) was acquired (Omnicam, Dentsply Sirona). This optical scan at baseline included the positions of the anterior teeth in maxilla and mandible, the surrounding gingiva, as well as the TRD. Then, the device was removed from the mouth. Over a period of several days, the device was repeatedly inserted and new intraoral scans were performed (in total n=10). The scans were exported as STL files, and aligned using best-fit algorithms (Geomagic Design X, 3D-Systems, Rock Hill, U.S.A.). In pair-wise comparison between reference and each follow-up scan, position changes of maxillary and mandibular soft and hard tissues were measured. Thus, accuracy of reproducing a specific maxillomandibular relation was analyzed by calculating root mean square (RMS) differences between initial (reference) and follow-up scans. Differences were statistically analyzed using Student’s t tests at a significance level of 0.05 (SPSS v 25, IBM, Armonk, United States).

## Results

### Applicability and Clinical Results

**Table 2** summarizes clinical characteristics and acute toxicity of the 10 patients treated with the novel TRD design. In all patients, a pre-fabricated device in correct size was available and customization was possible. All patients were able to insert and remove the device on their own during the entire radiation period. All TRDs remained undamaged until the end of radiotherapy. Acute treatment related toxicities were assessed regularly during and after radiation treatment. None of the patients developed a severe form of mucositis (grade III or IV).

**Table 2:** Patient and treatment characteristics and acute treatment-related toxicity (n = 10 patients).

Parameter	Count (%) or median (range)
Patient characteristics	
Age	54 (22–79)
Gender	
Female	4 (40)
Male	6 (60)
Eastern Cooperative Oncology Group (ECOG) status	
0	4 (40)
1	6 (60)
Tumor site	
Lip and oral cavity	4 (40)
Oropharynx	2 (20)
Nasal and paranasal sinus	4 (40)
Tumor stage	
T1	1 (10)
T2	1 (10)
T3	2 (20)
T4	6 (60)
Treatment characteristics	
1.1.1.1 Total dose of irradiation [EQD2]	70 (48–80)
1.1.1.2 Intensity-modulated radiation therapy (IMRT)	6 (60)
Proton therapy	2 (20)
IMRT + C12-boost	2 (20)
Acute toxicity	
Radiation dermatitis °I	5 (50)
Oral mucositis °II	4 (40)
Oral mucositis °I	3 (30)
Radiation dermatitis °II	2 (20)
Xerostomia °I	2 (20)
Dysphagia °I	2 (20)
Dysphagia °II	1 (10)
Dysgeusia °1	1 (10)
Xerostomia °II	1 (10)
Xerophthalmia	1 (10)

### Volumetric Analysis of Tongue Retraction

**Figure 3:**
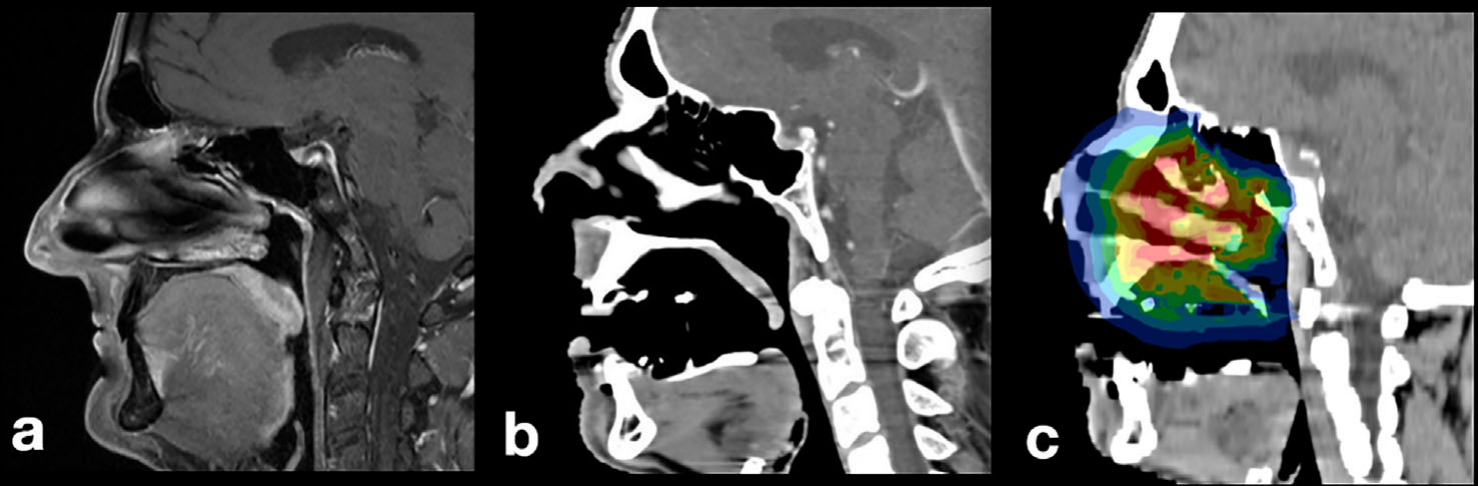
Patient with pleomorphic sarcoma of the nasal sinus: **(A)** diagnostic MR imaging without TRD, **(B)** baseline planning CT with incorporated TRD: the tongue is displaced to a caudal position, **(C)** irradiation plan without involvement of mandibular soft or hard tissues.

An effective tissue retraction and tongue displacement was achieved. With the TRD placed intraorally, substantially less healthy tissue and risk structures were present within the radiation field compared with diagnostic MRI. Usually, the tongue is in direct vicinity to the palate (**Figure 3A**). As a result of TRD use, a volume of air inside the oral cavity was measured averaging in 37.5 cm^3^ ± 23.6 cm^3^, indicating a substantial spacing effect (**Figures 3B, C** **)**.

### Reproducibility of Maxillomandibular Relation

**Figure 4:**
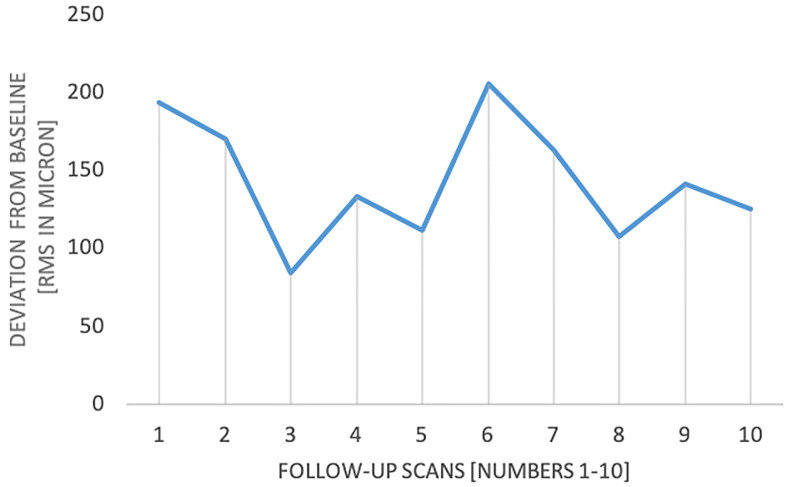
RMS differences between initial reference and follow-up scans.

Mean geometric deviation between reference and follow-up scans was 98.1 µm ± 29.4 µm RMS (max: 205.4 µm, min: 84.3 µm). Significant differences between the follow-up scans were found (p < 0.001), indicating statistically relevant deviations between the ten repetitions (**Figure 4**). However, after 10 repetitions, no material wear was recognized. Consequently, no apparent trend regarding a longitudinal decrease in accuracy was detected.

## Discussion

The 3D-printed TRDs tested in this study can be recommended for further scientific and clinical application. CAD/CAM technology proved useful for simplification of the traditional workflows. The devices were tolerated well by all tested patients over the entire irradiation period. No technical complications occurred. TRDs displaced the tongue by 37.5 cm^3^ ± 23.6 cm^3^. This has been shown to be beneficial regarding dose distribution and toxicity (14). Additionally, the TRDs limited daily inter-jaw position changes to a mean value of approximately 100 µm RMS.

For the TRD design presented here, the concept of customizing pre-fabricated structures was selected over producing fully individual appliances for each patient. This decision was based on a study which compared fabrication time and accuracy of fit of two fully individual TRD types based on i) segmented CT scans and ii) optical stone models scans (13). Regarding fabrication time, CT segmentation alone required, on average, 40 min, while optical scanning and model registration required a minimum of approximately 20 min. It has to be taken into consideration, that in both workflows the TRDs still need to be designed—on an individual basis. When adding the time for device design and for on-demand fabrication, fully individual TRDs seem inferior from the aspect of cost-efficiency.

Regarding accuracy of fit, it is generally possible to accommodate fully individual TRDs to the patients’ teeth (13). The optical scan method was significantly superior to the CT segmentation method. This result is not surprising: Optical scans of stone models are the gold standard for tooth surface digitalization in restorative dentistry. Reconstruction of tooth surfaces using three-dimensional imaging is substantially less accurate. In a previous study, geometric accuracy of tooth surfaces segmented from three-dimensional imaging (cone-beam computed tomography and MRI) was compared with optical scans. Deviations of between 102 to 261 µm RMS between imaging-based segmentations and optical scans were found (“segmentation errors”) (15). Additional errors will inevitably occur due to inaccuracies of the 3D-printing process. However, for the design presented here, neither segmentation errors nor manufacturing inaccuracies will affect fit. Segmentation is not necessary and 3D-printing inaccuracy were compensated by the customizing procedure using silicone impression material.

When adequately designed, TRDs can provide rigid inter-jaw fixation, which is a prerequisite for effective tongue displacement. Mean geometric deviation between reference and follow-up scans was approximately 100 µm RMS. For contextualization, the habitual intercuspation of fully dentate patients can be located with an accuracy of around 40 µm (16). In consequence, a full natural dentition is still 2.5 times more accurate in reproducing the maxillomandibular relation than the TRDs tested here.

This higher accuracy is probably caused by the use of rather flexible silicone material for adaption, in comparison to the hard tooth enamel. Nevertheless, for irradiation purposes, TRDs might represent a substantial improvement especially when adding the immobilization mask. No apparent trend regarding a longitudinal decrease in accuracy was detected. However, clearly, there will be an effect of dental status: The fewer teeth are available for TRD stabilization, the lower the accuracy in reproducing the maxillomandibular relation. In this study, only one fully dentate patient was evaluated. However, in the anterior mandible and maxilla, stabilizing silicone needed to be reduced to allow for intraoral scanning. Effectively, the TRD was supported by premolars and molars only, which resembles a partially edentulous patient.

Our TRD design allows for bi-lateral tongue displacement [e.g. for unilateral tonsil or tongue base carcinoma (17, 18)], caudal tongue displacement [e.g. for nasopharyngeal and palate tumors (13, 17, 19), or tongue carcinomas (20, 21)] and lip- and cheek-spacing [e.g. tumors of the buccal mucosa (13)]. One additional function is the possibility of ventral displacement of the tongue (i.e. tongue-out position). Radiation therapy in the head and neck area can cause swallowing difficulties depending on the radiation dose (22). Kil et al. described that a tongue-out position can reduce the radiation dose to the swallowing organs and thus possibly reduce side effects like dysphagia (6). However, ventral tongue displacement is limited by the connection bar (**Figure 1**, yellow structure). Potentially, a greater tongue displacement would be advantageous. However, it is unclear whether patients can sustain a more extended (more tiring), tongue-out position over the entire irradiation time. In this context, it is important to instruct the patient during customization to protrude the mandible, which supports the anterior displacement of the tongue base (6).

Tissue retraction may also reduce xerostomia if salivary glands are spared from radiation (e.g. with tongue carcinoma) (23). Xerostomia results from an impaired function of the major and minor salivary glands (24) with a relevant prevalence between 30% and 60% despite conformal IMRT (25, 26). It is a main cause of radiation caries (27) and therefore of tooth loss—with subsequent consequences, such as impaired chewing performance, speech ability, and quality of life (28). Apart from the major salivary glands, minor salivary glands are found in the entire oral cavity (25). Although they contribute to only about 10% of the total saliva flow (29), their mucous secretions are of great importance for the lubrication and protection of oral tissue (30). Increasing the distance of healthy tissue from the irradiation site can only be beneficial to reduce xerostomia.

Several important limitations of the TRD-design need to be addressed. Our semi-customization approach requires manual skills for TRD selection and adaptation. Since the TRDs cover the complete dentition and are made of one piece, a sufficient mouth opening of at least 20 mm is necessary. We have, nevertheless, decided in favor of the presented design as mouth opening increases the upper airway space (31). Therefore, breathing is facilitated during radiotherapy. Three sizes (small, medium, large) were sufficient to accommodate all patients. Extending the TRD size range is an option for the future. Gag reflex was another crucial limitation during the design process. Tongue depression would be more effective if the TP part was extended even further in posterior direction—at the cost of increasing patients` discomfort.

Therefore, the TP design was a compromise between effective displacement and patient tolerance. In addition, the TRD is customized prior to treatment planning. This process should therefore be organized in close cooperation of dentists and radiation oncologists with expertise in head and neck cancer.

In the current analysis, acute RIOM of grade I or II occurred in seven of 10 patients (70%) but none of the patients developed a severe form of acute RIOM (grade III or IV). This circumstance could indicate that tissue retraction had a beneficial effect on acute toxicity. However, these data must be interpreted with care due to the low number of patients. Prospective randomized clinical trials over longer follow-up time are merited. Here, the effects on acute and long-term toxicity, overall patient survival, quality of life, taste impairment, salivary flow rate, radiation caries and other parameters must be further investigated.

## Conclusion

The present results underline the high potential of a novel method for 3D-printed TRDs for radiotherapy in the head and neck area. TRDs were tolerated well by all tested patients. Reproducibility of maxillomandibular relation was high using a tooth-borne design. To further evaluate the potential clinical benefits of the developed TRDs, a randomized prospective phase II trial was initiated and registered under ClinicalTrials.gov, NCT04454697, on July 1^st^ 2020.

## Data Availability Statement

The datasets generated for this study cannot be made publicly available since national legislation and the terms of study ethics approval do not allow dataset sharing outside of the institutions participating in the analysis.

## Ethics Statement

The retrospective study involving human participants was reviewed and approved by Ethics committee of the Heidelberg University Hospital. Patient confidentiality was assured by anonymizing patient data and any identifying informations were removed.

## Author Contributions

CH, FSS, TH, LC, and SA developed and planned this analysis under the supervision of PR, KH, and JD. CH, MS, and FSS were responsible for the design, fabrication, and adaptation of the TRDs. TH, KL, CH, FSS, TH, and SA: data curation, analysis, investigation, validation, methodology, visualization, writing—original draft, writing—review, and editing. LC and MS: data curation, investigation, visualization, writing—review, and editing. JM, TM, and KZ: data curation, investigation, validation, writing—review, and editing. PP, KH, CF, PR, and JD: validation, supervision, writing—review, and editing. All authors contributed to the article and approved the submitted version.

## Funding

The authors acknowledge financial support within the funding program of the National Center for Tumor diseases (NCT) Heidelberg, Germany.

## Conflict of Interest

JD and SA Accuray International Sàrl outside the submitted work. SA received grants from Novocure outside the submitted work. JD and SA received grants from Merck Serono GmbH and Astra Zeneca GmbH outside the submitted work. SA holds shares in Novocure GmbH, Actinium Pharmaceuticals and Telix Pharmaceuticals. JD received grants from CRI – The Clinical Research Institue GmbH, View Ray Inc., Accuray Incorporated, RaySearch Laboratories AB, Vision RT limited, Astellas Pharma GmbH, Solution Akademie GmbH, Ergomed PLC Surrey Research Park, Siemens Healthcare GmbH, Quintiles GmbH, Pharmaceutecal Research Associates GmbH, Boehringer Ingelheim Pharma GmbH Co, PTW-Freiburg Dr. Pychlau GmbH, Nanobiotix A.A. outside the submitted work.

The remaining authors declare that the research was conducted in the absence of any commercial or financial relationships that could be construed as a potential conflict of interest.

## Abbreviations

CAD, Computer aided designing; CAM, Computer aided manufacturing; TRD, Tissue retraction device.
